# Professional Development in Autism and Multilingualism for Behavior Analysts: A Randomized Waitlist Control Trial

**DOI:** 10.1007/s10803-025-06730-1

**Published:** 2025-02-14

**Authors:** Melanie R Martin Loya, Hedda Meadan, Xun Yan

**Affiliations:** 1 University of California Davis, MIND Institute, Sacramento, USA; 2 University of North Carolina at Charlotte, Charlotte, USA; 3 University of Illinois Urbana Champaign, Urbana, USA

**Keywords:** Autism, Multilingualism, Bilingualism, Professional development, Behavior analysis

## Abstract

**Purpose:**

Autistic children and their families from heritage-language-speaking homes are underrepresented in empirical research and would benefit from receiving care tailored to their linguistic and cultural needs. Board Certified Behavior Analysts (BCBAs) commonly support autistic children in the United States but have reported limited knowledge and training in how to support bilingual children, their families, nor the bilingual staff who support them. The following research question was addressed: Do BCBA leaders in autism care who complete an asynchronous online professional development training demonstrate (a) increased knowledge, (b) improved self-efficacy, and (c) improved attitudes toward supporting multilingual Applied Behavior Analysis (ABA) staff and recipients of multilingual ABA services compared to BCBA leaders in a waitlist-control group?

**Method:**

Part of a larger sequential exploratory mixed methods study, this randomized waitlist control trial measured the impacts of a professional development training related to autism and multilingualism in ABA care on BCBAs’ knowledge, self-efficacy, and attitudes. Social validity data were also collected and analyzed using descriptive statistics.

**Results:**

The training significantly increased the intervention group’s knowledge and reported self-efficacy compared to participants in the waitlist control group but had no significant effect on participants’ attitudes. In addition, participants perceived the training as socially valid.

**Conclusion:**

Implications highlight the need for more attention on professional development research for autism providers, to examine pre-service training, and for clinical leaders to examine their workplace environment to promote effective and fair practices.

**Supplementary Information:**

The online version contains supplementary material available at 10.1007/s10803-025-06730-1.

Autistic children in the United States (U.S.) from multilingual or heritage-language-speaking homes are an underrepresented population in autism research (Steinbrenner et al., 2022) and encounter barriers to accessing evidence-based autism services (Lim et al., 2021). A *multilingual person* is defined as one who has a spectrum of expressive and receptive abilities in two (or more) languages (The American Speech-Language-Hearing Association [ASHA], 2023). Aligned with a strengths-based framework, the term *multilingual autistic children* will be used to refer to autistic children who are regularly exposed to a heritage language throughout their daily life, regardless of their level of communicative abilities (e.g., this includes emerging communicators, see ASHA, 2023 and Ohashi et al., 2012). A *heritage language* is defined as any language other than the dominant language in a given society; in the context of the U.S., this refers to any language other than English (Kelleher, 2010). Multilingual autistic children may benefit from receiving culturally and linguistically tailored evidence-based care (Baires et al., 2023; Trelles & Castro, 2019; Vargas Londono et al., 2024) and the ability to learn a second language or maintain their heritage language(s) has been reported as an area of great value to autistic individuals (Davis, 2023; Digard et al., 2020, 2022; Nolte et al., 2021).

Research on the impacts of multilingualism on autistic children is nascent, but there is some evidence that growing up multilingual may benefit autistic children in executive functioning (Montgomery et al., 2021; Ratto et al., 2020, 2022; Sharaan et al., 2022) and social and language skills (Beauchamp et al., 2020; Siyambalapitiya et al., 2022; Zhou et al., 2019). Researchers who employed single case methodology to examine multilingualism in children with autism or other developmental disabilities reported mixed results. In preference assessments, some children strongly preferred their families’ heritage language over English (e.g., Kunze et al., 2019), while others indicated equal preference across languages (e.g., Padilla Dalmau et al., 2011). In addition, children sometimes displayed higher rates of challenging behaviors when presented with tasks in one language over another (e.g., Rispoli et al., 2011), and other children displayed minimal to no differentiation across language conditions when presented with tasks (e.g., Neely et al., 2019). Lastly, some learners engaged in higher rates of correct responses to academic and language tasks in their heritage language conditions (e.g., Lang et al., 2011), while others had improved responses in English-only conditions (e.g., León & Rosales, 2018). Varied results from the single-case studies highlight the importance of individualized assessment and intervention practices for multilingual autistic children and demonstrate the important role of the professionals who guide educational and clinical treatment plans for autistic children.

## Knowledge Gaps and Workforce Challenges in Dual Language Instruction

While there is a wealth of research and support for dual language^1^ instructional practice for children without disabilities (Soto-Boykin et al., 2024), our knowledge of best practices to support multilingual autistic children in educational or clinical settings is limited due to underrepresentation in research. However, we know that children are best served when assessed using all the languages within their repertoire (Soto-Boykin et al., 2024), which is supported by the heterogeneity of results from single case studies related to autism and multilingualism (i.e., children will have better outcomes when their education and care is tailored to their specific needs). In addition, one study has been published that examines the outcomes of dually identified autistic students in one California district (Castellón et al., 2024). *Dually identified* is defined as a student who receives services under the Individuals with Disabilities Act (e.g., autism) and is also identified as an “English Learner” (i.e., a multilingual child). Castellón and colleagues (2024) found that dually identified autistic students who were primarily placed outside of the general education classroom achieved English proficiency later and at much lower rates than dually identified autistic students who spent more time in general education, which supports calls to improve educational and clinical settings that serve multilingual autistic children. Unfortunately, many autistic children are unable to access multilingual learning opportunities (Davis et al., 2022), and parents of autistic children have reported that educators and service providers often advise them to use only one language with their children (Durán et al., 2022), which is in stark contrast with the empirical literature and current recommendations (Gilhuber et al., 2023).

Another persistent challenge to improve autism care for multilingual children and their families relates to the professional workforce. Demographics of professionals who routinely serve autistic populations in the U.S., such as Board Certified Behavior Analysts (BCBAs) or Speech Language Pathologists, are not reflective of the communities they serve (i.e., they belong to a workforce that is largely White, monolingual, and female; ASHA, 2023; Behavior Analyst Certification Board [BACB], n.d.). In addition, BCBAs have reported limited knowledge and receiving no training in how to support multilingual populations (Beaulieu et al., 2019; Martin Loya & Meadan, 2024a, b); however, we know that providers in ABA can quickly and effectively gain skills to better support linguistically diverse families, such as learning to work with interpreters (Vazquez et al., 2024). We also know that autistic children and their families who primarily speak languages other than English form a population that is projected to grow (Vespa et al., 2020), who experience disparate access to high-quality educational opportunities (Barrera-Lansford & Sánchez, 2024; Burke et al., 2021; Castellón et al., 2024), and encounter barriers when accessing autism specialist support (Lim et al., 2021). This is a major issue of inequity in practice and represents a significant gap in the research literature. Therefore, the present study addresses the need for more professional development to enhance autism professionals’ knowledge, self-efficacy, and attitudes related to supporting multilingual autistic children, their families, and the multilingual direct care providers who support them. Specifically, this study focuses on U.S.-based BCBAs who provide services to autistic populations and their families.

## Knowledge, Self-Efficacy, and Attitudes of BCBAs

BCBAs are graduate-level clinicians with expertise in the science of behavior change, and most of them live in the U.S. and practice with autistic populations (BACB, n.d.). In addition, BCBAs must complete ongoing Professional Development (PD) in the form of Continuing Education Units (CEUs) to remain abreast of new developments in the field and maintain their certification (BACB, 2023a). Research on behavior analysts’ professional development and continuing education is limited. For example, a recent literature review explored the landscape of asynchronous learning of behavior analytic practice, but none of the studies included behavior analysts as participants (Gerencser et al., 2020), and most published research related to supervision in behavior analysis is conceptual, rather than empirical (Kranak et al., 2023). However, we know that most behavior analysts tend to accrue their required CEUs online (e.g., webinars or other virtual events; Kranak et al., 2022), making online asynchronous training an important PD modality for BCBAs. Asynchronous training without additional components such as coaching can positively impact providers’ content knowledge and practice behaviors, even when the training is very short. For example, a one-hour asynchronous online training without coaching increased healthcare professionals’ knowledge and self-reported comfort in new evidence-based practices immediately after training and maintained at a two-month follow-up (Raffoul et al., 2022), and another one-hour training evaluated using a cluster Randomized Control Trial (RCT) demonstrated changes in physician knowledge and practice behaviors to the benefit of their patients (Verbist et al., 2014). Most published studies that examined one-hour PDs were in the healthcare field (e.g., Cai et al., 20r15), and unfortunately, the efficacy of a short one-hour asynchronous training has not been explored among BCBAs despite the popularity of such modality.

Self-efficacy is a psychological concept that refers to an individual’s belief that they can enact change or take action to accomplish a goal (Bandura, 1997). The importance of self-efficacy and its association with various constructs is well-studied and has been measured across cultures and environments; for example, a meta-analysis with over 100 studies examined leadership work performance (Harari et al., 2021), a meta-analysis of 57 studies demonstrated a link between low self-efficacy and burnout among educators and healthcare workers (Shoji et al., 2016), and a 40-year research synthesis of 165 studies showed self-efficacy’s impacts on teachers’ classroom processes, student academics, and teacher well-being (Zee & Koomen 2016). To date, only one study was found that examined self-efficacy related to ABA and autism; however, not all of the participants were BCBAs (Marshall et al., 2023). Thus, self-efficacy is a well-studied construct that predicts workplace performance in human service fields similar to ABA, and this concept has room for exploration within ABA and autism care.

Attitudes are another widely studied psychological construct that are based on beliefs and refers to an individual’s tendency toward particular judgments that may predict behaviors (Eagly & Chaiken, 2007; Pettit, 2011). Additionally, attitudes have directionality; they always target a particular person, place, ideology, or practice. Associations exist between individuals’ attitudes and behaviors across environments relevant to BCBAs and other autism service providers, including within classrooms (Fallon et al., 2022; Kim, 2021; Locke et al., 2019) and in clinical decision-making (Easton & Verdon, 2021). For example, in special education classrooms for autistic children, teachers’ decisions to implement or not implement evidence-based practices were influenced by their attitudes (Locke et al., 2019), and SLPs with negative attitudes toward “non-standard” uses of the English language (e.g., Black or African American English) were found to engage in more discriminatory clinical decision making (Easton & Verdon, 2021). Thus, the attitudes disability professionals hold can have significant impacts on the lives of children with disabilities and their families, and attitudes are a construct that should be directly addressed in PD training to promote improved and more equitable practices.

Overall, there is an understanding that behavior analysts benefit from ongoing PD opportunities, and there is a need for organizational leaders to enact change to support ABA providers. Additional research on professional development for behavior analysts who support autistic children is needed. Therefore, a randomized waitlist control study was conducted to measure the impacts of a short asynchronous PD training on the knowledge, self-efficacy, and attitudes of BCBAs in autism care toward multilingualism and supporting multilingual ABA staff, learners, and their families.

## Method

This study aimed to improve BCBA leaders’ knowledge, self-efficacy, and attitudes related to supporting multilingual staff and families to enhance care for recipients of multilingual ABA (i.e., multilingual children and their families who receive ABA services). This is important because recipients of multilingual ABA are more likely to be members of underserved and marginalized groups, and behavior analysts have reported limited knowledge and PD opportunities in this area (Beaulieu et al., 2019; Martin Loya & Meadan, 2024a). This project was guided by the following research question: Do BCBA leaders in autism care who complete an asynchronous online professional development training demonstrate (a) increased knowledge, (b) improved self-efficacy, and (c) improved attitudes toward supporting multilingual ABA staff and recipients of multilingual ABA services compared to BCBA leaders in a waitlist-control group?

### Design

This study was part of a larger exploratory sequential mixed methods experimental design (Creswell, 2022; Greene, 2007) that addressed multiple research questions. This article focuses on the waitlist RCT intervention.

### Researchers’ Identities

All authors speak at least two languages and have international personal and professional experiences. The first two authors are BCBAs and have personal, clinical, and academic experiences related to autism and multilingualism. The third author is an expert in linguistics and quantitative research methods.

### Recruitment and Participants

Participants were recruited to join the study using three methods: (1) sharing flyers in public social media spaces that target behavior analysts, (2) recruitment via professional ABA organization listservs, and (3) chain-referral sampling (i.e., snowball sampling). The population targeted for recruitment was BCBA leaders in autism care. They were defined as (a) BCBAs in good standing per the BACB, (b) having completed a BACB-approved 8-hour supervisor training, (c) being at least one-year post-certification, (d) primarily serving autistic children and their families, (e) currently providing mentorship or supervision to other ABA staff or students of ABA, and (f) aged 18 or older and live and work in the United States. An a priori power analysis determined that 128 participants were needed to achieve power at 0.80 with a medium effect size (Faul et al., 2009). However, the study was ultimately underpowered. In total, 160 individuals filled out the Google form screener, and 154 were eligible to participate. Reasons participants were not eligible included not being BCBAs and/or not working primarily with autistic populations. The 154 eligible individuals were invited to fill out the pre-tests in Qualtrics (i.e., consent, demographic form, and pre-measures), of which 122 individuals completed all required pre-test questions. The 122 individuals were randomly assigned to an intervention or waitlist control group across three cohorts. The final number of participants included for analysis was 43 in the intervention group and 47 in the waitlist control group (*N* = 90). Anecdotally, some participants reported not completing all of the study components due to a lack of time or forgetting to complete the tasks. The 90 participants were a diverse group of behavior analysts who spoke 14 distinct languages. Monolingual English speakers were most common (*n* = 61), followed by multilingual speakers of Spanish (*n* = 15), Arabic (*n* = 4), French (*n* = 3), and Korean (*n* = 3). Participants were also from 25 different U.S. states, with most participants from California (*n* = 15), New Jersey (*n* = 14), Ohio (*n* = 9), Washington (*n* = 8), Illinois (*n* = 7), and Texas (*n* = 5). Most participants reported receiving no training in how to support multilingual ABA staff (*n* = 85) or recipients of multilingual ABA (*n* = 72). Finally, most participants identified as female (*n* = 89); only one male completed all study components and was included in the analyses. See Table 1 for additional demographic information about participants and descriptive statistics.  

**Table 1:** Participant Demographic Characteristics

	Intervention			Waitlist Control			All Participants	
	n/*Mean*	%/*Range*	n/*Mean*		%/*Range*		n/*Mean*	%/*Range*
**Language Ability** ^**a**^	*n* = 43			*n* = 47			*N* = 90	
Monolingual English	30	69.8		31		66	61	67.8
Bilingual	11	25.6		13		27.7	24	26.7
Multilingual	2	4.7		3		6.4	5	5.6
**Age in Years**	*38.2*	*26–69*		*36.5*		*27–65*	*39.5*	*26–69*
**Yrs. Experience in ABA**	*12.7*	*3–38*		*13.1*		*3–34*	*12.1*	*3–38*
**Certificate Level**								
BCBA – Masters	40	93		42		89.4	82	91.1
BCBA – Doctoral	3	7		5		10.6	8	8.9
**Race/Ethnicity**								
White or European American	27	62.8		33		70.2	60	66.7
Hispanic or Latino	8	18.6		8		17	16	17.8
Asian	2	4.7		3		6.4	5	5.6
Black or African American	2	4.7		1		2.1	3	3.3
Middle Eastern or North African	2	4.7		1		2.1	3	3.3
Two or More Identities	2	4.7		1		2.1	3	3.3
**Training (Not) Received**								
No Training to Support Multilingual Staff	41	95.3		44		93.6	85	94.4
No Training to Support Multilingual Families	37	86		35		74.5	72	80
**Connections to Autism** ^**b**^								
Close Family Member	19	44.2		24		51	43	47.8
Work only	22	51.2		18		38.3	40	44.4
Close Friend	15	34.9		14		29.8	29	32.2
Autistic/Person with Autism	6	14		4		8.5	10	11.1
Prefer not to share	1	2.3		2		4.2	3	3.3

Note: ^*a*^Bilingual means participants could communicate in two languages (including English), and multilingual means participants could communicate in three or more languages. ^*b*^Percentage may not equal to 100 because some participants had multiple identities and relationships related to autism

#### Procedures

Interested participants accessed a Google screening form (see Supplemental Information [SI]) by following the URL on the recruitment flier, email, or social media post. Upon completing the screening form, all potential participants were individually screened by the first author and approved to participate by cross-checking their reported demographics with the public database of BCBAs hosted by the BACB. Participants were screened on a rolling basis between November 2023 and February 2024 and admitted into the intervention across three cohorts. All cohorts included a ten-day pre-test window, a ten-day intervention/wait period, and a ten-day post-test window. To combat attrition, the waitlist control groups were provided access to watch the PD immediately upon completion of the post-measures, and they were not required to wait until the full 10-day window was complete.

Once approved, each cohort was sent an individualized Qualtrics link that included consent, demographic forms, and the pre-measures. The pre-measures included (a) a researcher-created knowledge assessment about autism and multilingualism, (b) a self-efficacy scale of participants’ perceived ability to put their knowledge into action in their places of work, and (c) an attitude toward languages scale. Pre-measures were identical to post-measures. Participants in each cohort were then randomly assigned to the intervention group or a waitlist control group using an online random generator. The independent variable was a 50-minute PD targeted to BCBA leaders in autism care to improve their understanding of multilingualism, autism, and strategies for supporting multilingual direct-care staff. More details about the intervention can be found in the section below.

After the intervention, both groups completed a social validity questionnaire to assess the PD’s acceptability and feasibility. All aspects of this study were conducted online, and the total time commitment for participants was approximately one and a half hours. As a token of appreciation, twenty e-gift cards of 50-dollar value were raffled to participants who chose to enter their email addresses after the study. In addition, the successful completion of the study included one Ethics CEU for BCBAs.

#### Intervention Development and Delivery

The PD training was informed by Adult Learning Theory and *just-in-time* training, a method wherein the learner seeks out training based on their own needs and desires (Trivette et al., 2009). PD for behavior analysts often falls under the category of *just-in-time* training (e.g., attending webinars or lecture-based conference presentations; Kranak et al., 2023). The PD in this study is considered just-in-time training, and its development was informed by empirically based recommendations, including (a) introducing information and key terminology (i.e., the PD starts with defining key terms and necessary legal information such as the Individuals with Disabilities Education Act and the Americans with Disabilities Act), (b) illustrating the information using active engagement (e.g., required pop-up questions and requiring participants remain on the website for a set amount of time), and (c) learner assessment of mastery (e.g., affirmative or corrective feedback from the pop-up questions). Extant empirical literature and qualitative findings from the first phase of the larger mixed-methods study informed the content of the PD training, including (a) useful terminology and establishing the importance of heritage languages, (b) a review of the empirical literature on autism, multilingualism, and ABA, (c) legal and ethical considerations (e.g., BACB Ethics Code, 2020), (d) reported experiences and needs of multilingual behavior analysts (Martin Loya & Meadan, 2024a, b), and (e) action steps to improve ABA practice.

Some major takeaways from the training included highlighting how multilingual autistic children are likely to have language preferences and that behavior analysts should actively measure and honor those preferences as much as possible. The training also emphasized that children may have improved clinical outcomes when receiving heritage language care compared to English-only. Additionally, it stressed the importance of specifically assessing family preferences and values related to language use (e.g., if the child has extended family members who speak a different language, and the caregivers want the child to know basic greetings in that language to enhance their familial connections). Additional details about the training can be found in the SI and a forthcoming article.

#### Data Collection

Data sources for this study included (a) a demographic questionnaire, (b) pre- and post-intervention measures, and (c) a post-intervention social validity questionnaire.

#### Measures and Instruments

**Demographic Questionnaire.** The demographic questionnaire included questions about racial and ethnic identity, generation in the U.S., language(s) spoken, work and certification history, and personal connections to autism outside participants’ professions.

**Knowledge Assessment.** The knowledge assessment was researcher developed and informed by the most recent empirical evidence related to autism and multilingualism and included topics such as (a) multilingual autistic child outcomes (Gilhuber et al., 2023), (b) experiences of linguistically diverse caregivers of autistic children (Durán et al., 2022; Papoudi et al., 2021), (c) multilingual autistic adults’ experiences and preferences (Digard et al., 2020, 2022; Nolte et al., 2021), and (d) multilingual behavior analysts’ reported experiences and needs (Martin Loya & Meadan, 2024a, b). This assessment was directly connected to the content of the PD. The final assessment included fourteen questions and took about ten minutes to complete. Possible scores ranged from zero (no questions were answered correctly) to 19 (all answers were correct). Cronbach’s alpha was not used to measure the internal validity of the knowledge assessment because the assessment included multiple constructs.

**General Self-Efficacy Scale (GSE).** The GSE from Schwarzer and Jerusalem (1995) was utilized for the present study to measure participants’ perceived self-efficacy (Bandura, 1997). The GSE has 10 items using a four-point scale ranging from 1 = Not at all to 4 = Exactly true. Higher scores in the GSE indicate higher perceptions of self-efficacy. The GSE was adapted for the present study by adding two Likert scales per item, with one scale about participants’ experiences with heritage-language-speaking families and the other scale about direct-care staff who work with heritage-language-speaking families (i.e., the final number of items equals 20). This was adapted in consultation with community members to capture differences that may be experienced by behavior analysts who work in a range of environments as they support recipients of multilingual ABA and staff. The items were presented randomly per the developers’ recommendations (Schwarzer & Jerusalem, 1995) and took approximately ten minutes to complete. The adapted scale used in the present study had satisfactory internal consistency, with a Cronbach’s alpha of 0.92.

**Language Attitudes of Teachers Scale - Revised (LATS-R).** The LATS has been revised multiple times (see Byrnes & Kiger, 1994 and Flores & Smith, 2009) and was updated more recently using a new factor structure in alignment with current multilingual education literature (Cho et al., 2023). For the present study, we adapted the 2023 LATS-R by removing two irrelevant items, replacing the word “teacher” with “behavior analyst,” and adapting examples to focus specifically on autistic students. For example, “Teachers should modify their instruction for their students’ cultural and linguistic needs” was adapted to: “Behavior analysts should modify their instruction for their students’ cultural and linguistic needs.” The final adapted LATS-R used in this study had an acceptable internal validity with a Cronbach’s alpha of 0.78, had 11 items, and took less than ten minutes to complete. On this scale, a lower score indicated more open attitudes toward languages (e.g., marking ‘Strongly Agree’ to the item, *Behavior analysts should modify their instruction for their students’ cultural and linguistic needs* results in a score of 1). Thus, scores could range from 11 (most favorable attitudes toward multilingualism) to 55 (least favorable attitudes toward multilingualism).

**Social Validity Questionnaire.** The social validity questionnaire had three parts based on Wolf’s social validity model (1978): acceptability, feasibility, and perceived effectiveness. The questionnaire was researcher-developed and was informed by relevant recent social validity literature (e.g., Larson et al., 2020; Snodgrass et al., 2022). All sections had five-point Likert scales ranging from zero (strongly disagree) to five (strongly agree) and ended with four optional open-ended questions for participants to provide additional comments. This measure had high internal validity with a Cronbach’s alpha of 0.84 and took less than ten minutes to complete. All participants in the intervention group completed the social validity questionnaire (*n* = 43), but some participants in the waitlist control group did not (*n* = 7), resulting in 83 completed social validity questionnaires. Only the Likert scale results are showcased in this article. See SI to see all the measures and a study design table.

#### Community Involvement: Reviewing the PD and Measures

Academics and community members reviewed the PD and all measures prior to implementation, and none of the community members were included in the RCT analyses. In addition, phase one of the larger mixed-method study included monolingual, multilingual, autistic, and non-autistic BCBA focus-group participants (*N* = 14) providing feedback on an initial outline of the PD, providing content validity prior to the PD being fully developed (please see forthcoming article for more information on the rest of the study). The first draft of the PD was reviewed by five additional behavior analysts, including doctoral and masters-level BCBAs, monolingual and multilingual BCBAs, and autistic and non-autistic behavior analysts who were not involved in phase one of the larger study. Feedback included suggestions to add more graphics, citations, and reorganization of recommendations. Their feedback resulted in minor edits throughout the training and the creation of a resource document that was shared with participants after they watched the PD.

Eight BCBAs, who were monolingual, multilingual, doctoral, and masters-level researchers and clinicians, reviewed the measures. Their feedback resulted in significant adjustments to the self-efficacy scale (e.g., adding an additional Likert scale per item and clarifying definitions). Minor clarifications were also added to the demographic questionnaires. The demographic question about individuals’ relationships to autism was crafted with feedback from an autistic PhD student in special education who was not a BCBA. No other substantial adjustments were made.

#### Data Analysis

RCT data were analyzed using SPSS version 29.0.2.0. First, descriptive statistics were calculated to check for normality and determine an appropriate method to examine the data​​. Then, Cronbach’s alpha was calculated for the attitude and self-efficacy scales to determine the internal validity of the measures. Next, depending on the nature and normality checks of the variables, the data were examined using either chi-square (non-parametric statistics) and independent sample t-tests (parametric statistics) to examine whether the intervention and waitlist control groups were statistically different across pre-measure scores, race or ethnicity, languages spoken, years of professional experience, or age. Once groups were determined to be the same, they were analyzed using multivariate analysis of variance (MANOVA). Specifically, a 2 × 2 factorial MANOVA was conducted with repeated measures within (Time 1 and Time 2) and between groups (intervention and waitlist control) as the independent variable. To measure treatment effects, the dependent variables included composite scores for each of the three measures (i.e., knowledge, self-efficacy, and attitude scales). Wilks’​​ 𝛌 was used as the global test to determine if there were any significant multivariate effects associated with time, group, and the interaction between the two. Then, univariate analyses were used to more closely examine significant differences to determine which dependent variables had different means across time and groups. Lastly, the social validity questionnaire was analyzed using descriptive statistics.

#### Quality Indicators for Quantitative Research

Various quality indicators were addressed in this study, including comparing intervention and control groups, calculation of internal consistency, the measurement of intervention effects, the use of previously validated measures, attention to implementation fidelity through the use of Qualtrics, and an a-priori power analysis (Gersten et al., 2005; Toste et al., 2023). In addition, attention to contextual fit was addressed by implementing the initial qualitative phase of the larger mixed methods study to develop the training, and social validity was measured post-intervention to evaluate acceptability and feasibility further.

## Results

This study focused on the impacts of a tailored PD training on behavior analysts’ knowledge, self-efficacy, and attitudes by employing an RCT design with intervention and waitlist control groups, followed by a social validity questionnaire. First, descriptive statistics were generated and are summarized in Table 2. Second, categorical demographic variables were tested for group differences using Chi-Square, resulting in no significant differences between the intervention and the waitlist control groups across language ability (i.e., monolingual or multilingual; *p =*.938), certification level (i.e., master’s or doctoral level; *p* =.716), race/ethnicity (*p =*.884), training received in supervising multilingual staff (*p* =.543), or training received in how to support heritage language speaking families (*p* =.196). Gender was not tested because only one male participated. In addition, independent samples t-tests were used to determine if there were significant differences between the groups in terms of age, *t*(88) = 0.967, *p* =.336, *d* = 0.204, and the number of years of professional experience in the field of ABA, *t*(88) = − 0.270, *p* =.79, *d* = − 0.057. No significant differences were found. There were also no significant differences between the groups on the knowledge pre-measure scores, *t*(88) = 0.325, *p* =.75, *d* = 0.069, self-efficacy pre-measure scores, *t*(88) = − 0.937, *p* =.35, *d* = − 0.198, nor attitudes pre-measure scores, *t*(88) = 0.321, *p* =.75, *d* = 0.068. Next, MANOVA assumptions were tested. Box’s Test was used to determine whether the covariance matrices of the dependent variables were equal across groups, and there was no significant violation (*p* =.392). Therefore, based on these variables, the intervention and waitlist control groups were determined not to be significantly different from each other, and MANOVA was determined to be an appropriate method to analyze differences within and between the groups.  

**Table 2:** Descriptive Statistics of Measures Across Groups

Measure	Intervention, *n* = 43, *M* (SD)		Waitlist Control, *n* = 47, *M* (SD)		F(1,88)	ηp²
	*Pre-*	*Post-*	*Pre-*	*Post-*		
Knowledge	*14.84* (2.91)	*17.44* (1.86)	*14.66* (2.26)	*15.91* (2.07)	7.508**	0.079
Self-Efficacy	*61.33* (7.48)	*64.65* (7.27)	*62.91* (8.52)	*63.62* (7.88)	4.659*	0.050
Attitude	*19.65* (5.42)	*18.95* (4.4)	*19.30* (5.01)	*19.89* (5.23)	2.849	0.031

*Note: The range of the possible composite scores for the knowledge assessment*,* self-efficacy scale*,* and attitude scale were 0–19*,* 20–80*,* and 11–55*,* respectively. F scores depict the interaction effect of group x time. Partial eta squared is depicted as* ηp²

*p < .05, **p < .01

Outcome data were analyzed using MANOVA with alpha = 0.05. Results indicated a non-significant main effect of group (𝛌 = 0.947, *F*[3, 86] = 1.617, *p* =.191, ηp² = 0.053), a significant main effect of the intervention across time (𝛌 = 0.552, *F*[3, 86] = 23.298, *p* < .001, ηp² = 0.448), and a significant interaction effect between group and time (𝛌 = 0.862, *F*[3, 86] = 4.602, *p* =.005, ηp² = 0.138). Because the analyses suggested significant main and interaction effects with at least one outcome variable, additional univariate analyses were conducted and are described below.

There was a significant effect for the interaction of group x time on knowledge, *F*(1, 88) = 7.508, *p* =.007, ηp² = 0.079, and self-efficacy *F*(1, 88) = 4.659, *p* =.034, ηp² = 0.050. These results indicate that there was a significant difference between intervention and waitlist control group scores as well as between pre- and post-tests. Lastly, there was no statistically significant interaction effect on the attitude scale, *F*(1, 88) = 2.849, *p* =.095, ηp² = 0.031. Overall, the attitude scale resulted in low power and no statistical significance. Please see SI for interaction plots for knowledge, self-efficacy, and attitude scales.

### Social Validity Results

The social validity questionnaire was analyzed with descriptive statistics which are summarized in Table 3. The majority of participants highly rated the PD’s importance and acceptability of goals and content, procedures, and perceived efficacy, despite the existence of one outlier in the intervention group who rated Questions 1–4 as “1”, noting they did not believe the training content, nor goals were appropriate. However, in an open-ended question, they reported that they “loved” the training. Overall, the highest-rated questions across groups were Question 2: I believe the content covered in this online training is important for advancing equity in ABA services (*M* = 4.85 out of 5), and Question 4: The content covered in this online training used appropriate and inclusive language (*M* = 4.91 out of 5). The lowest rated questions were Question 10: I feel more prepared to support dually identified children and families as a result of this training (*M* = 4.27 out of 5), and Question 11: As a result of this training, I plan to engage in at least one action to improve my or my organization’s practices related to bilingual staff and families (*M* = 4.56 out of 5).  

**Table 3:** Descriptive Statistics of the Social Validity Questionnaire Across Groups

Questions	Intervention		Waitlist Control		All Participants	
	*M*	*SD*	*M*	*SD*	*M*	*SD*
Q1	4.65	0.870	4.77	0.427	4.71	0.694
Q2	4.77	0.684	4.95	0.223	4.85	0.524
Q3	4.70	0.773	4.82	0.451	4.76	0.639
Q4	4.86	0.639	4.97	0.16	4.91	0.477
Q5	4.74	0.539	4.82	0.389	4.78	0.472
Q6	4.72	0.549	4.95	0.223	4.8	0.439
Q7	4.74	0.727	4.44	0.882	4.6	0.814
Q8	4.67	0.566	4.64	0.537	4.66	0.549
Q9	4.74	0.539	4.79	0.469	4.77	0.504
Q10	4.3	0.887	4.23	0.902	4.27	0.890
Q11	4.63	0.578	4.49	0.856	4.56	0.722

*Note: All questions were on a five-point Likert scale with 1 = Strongly disagree and 5 = Strongly agree. Questions 1–4 related to the importance of the goals and content*,* questions 5–7 related to the acceptability of procedures*,* and questions 8–11 related to the perceived efficacy of the PD*,* see SI for more details*

## Discussion

The purpose of the present study was to measure the impacts of a one-hour asynchronous PD on the knowledge, self-efficacy, and attitudes of BCBAs related to (1) supporting multilingual ABA staff and (2) supporting recipients of multilingual ABA services. This RCT study demonstrated a statistically significant increase in participants’ knowledge and self-efficacy after participating in the PD but no significant changes in participants’ attitudes. Additionally, participants reported high social validity of the training. This was the first study to measure the impacts of short asynchronous training on BCBA’s knowledge, self-efficacy, and attitudes related to multilingualism and autism, and highlights important areas to consider and target in future research and PD for autism-serving professions to improve equitable and culturally responsive evidence-based care.

The results align with existing research and suggest that tailored PD in the form of short asynchronous training can effectively increase participants’ knowledge and self-efficacy and is perceived by participants as socially valid (Raffoul et al., 2022). We know that behavior analysts routinely accrue their CEUs online (Kranak et al., 2022), and providers in healthcare fields have demonstrated increases in knowledge after receiving a one-hour asynchronous online training (Cai et al., 2015; Raffoul et al., 2022; Verbiest et al., 2014). Notably, in the current study, no impacts on participants’ attitudes were detected. A longer engagement with these topics may be needed to change attitudes and study limitations may have also contributed to the lack of change (see limitations section below). Overall, the PD was effective but should be viewed as a first step toward improving providers’ knowledge and self-efficacy toward multilingualism and autism to enhance their practices, and more work is needed to better understand attitudes. As evidenced by the two lowest-rated social validity questions, participants felt they learned new things and were eager to implement them. Still, they perhaps did not have all the tools needed to feel prepared to support families, nor were they necessarily able to identify a clear step to take to improve their own practices or those of their organization.

### Implications

This study has implications for practice, research, and policy. First, the majority (94.4%) of participants reported receiving no prior training related to autism and multilingualism. In practice, providers should seek out mentorship and community of practice opportunities to gain more skills related to supporting linguistically and culturally diverse children, family, and staff members. In the absence of structured training opportunities, providers are encouraged to seek out peer leaders (e.g., multilingual and multicultural providers) to provide additional support and guidance. However, providers are also cautioned to ensure they do not unfairly place additional uncompensated burdens on their multilingual colleagues, which has been reported as a challenge for multilingual autism providers (Martin Loya & Meadan, 2024b) and allied multilingual professionals such as educators (Amanti, 2019) and counselors (Pope et al., 2022). Bilingual providers are encouraged to self-advocate for necessary resources (e.g., translation services) and set boundaries with colleagues (e.g., establishing at the onset of a professional relationship if translation or interpretation are in their skillset, and if so, how they will be fairly compensated and scheduled to provide such services). Those in leadership positions in practice are also encouraged to engage in open conversation with their teams about the impacts of language and culture on staff, children, and families and take steps to improve their practice in alignment with recent recommendations for autism service providers such as BCBAs (Rosales et al., 2023a, b).

Implications for research highlighted in this study include the broad need for more exploration of multilingualism, autism, and professional development across disciplines. The professional development needs and training outcomes of BCBAs are likely to be different than those of allied professionals such as special educators, psychologists, occupational therapists, or speech language pathologists. Research that includes more members of the autism community, such as self-advocates, autistic providers, family members, and providers across disciplines, can paint a clearer picture of the professional development needs of autism providers who support multilingual autistic children and their families. With additional knowledge, we can take steps to ensure practices are effective, child- and family-centered, and in alignment with calls for more inclusive, neurodiversity-affirming, and effective autism research practices (Nicolaidis et al., 2019). Researchers are also encouraged to include practical elements in their research that may directly benefit providers whenever possible, such as coaching and providing individualized performance feedback (e.g., Chung et al., 2022), as well as engaging in open science practices and providing free resource documents, similar to recent recommendations for special education research (Toste et al., 2023).

Policy implications include exploring the training requirements of autism providers to ensure they are trained to work competently in a rapidly growing and diversifying world. While the new addition of cultural competency standards in the updated BACB Ethics Code (2020) and upcoming requirements for BCBAs to earn CEUs in Diversity, Equity, and Inclusion to take effect in 2027 (BACB, 2023b) are good first steps to support in-service BCBAs, the pre-service training of BCBAs should also include required training in cultural and linguistic diversity, similar to allied fields such as clinical psychology or speech language pathology (ASHA, 2023). Indeed, an additional implication of this work is for autism professionals in practice and research to work collaboratively within and across disciplines when supporting multilingual autistic children and their families or conducting research in this area. We know that multilingual autistic children and their families have been historically marginalized in the U.S. (Connor et al., 2016; Lim et al., 2021; Steinbrenner et al., 2022) and would benefit from a range of supports to address structural challenges such as service access barriers in education (Barrera-Lansford & Sánchez, 2024) and in clinical practice (Lim et al., 2021).

### Limitations

As with any study, this study had limitations that must be considered. The first limitation was a smaller sample size, which resulted in the study being underpowered which may have impeded our ability to draw conclusions. Participants in the intervention group did have improved attitude scores post-intervention, but the difference was not enough to reach statistical significance. Perhaps with a larger sample size, we may have been able to draw stronger conclusions about the impacts of the training on participants’ attitudes. Another potential limitation of the present study may have been the occurrence of social desirability bias (Fisher & Katz, 2000), where participants provide answers in alignment with what they believe their social group expects or prefers. While participants’ responses were de-identified, personal identifying information was collected as a requirement to provide participants with their CEU certificate. Participants’ offering of their personal information may have impacted their reported attitudes because the BACB Ethics Code prohibits discrimination (BACB, 2020). That is, participants across groups already leaned toward more accepting attitudes of multilingualism at the pre-test. It is unknown if the reported attitudes are reflective of population sentiment, and a fully anonymous survey that does not collect personal data may be better suited to better understand the attitudes held by behavior analysts and other autism providers toward languages. In addition, self-selection bias may be another limitation that impacted participants’ leaning toward more accepting attitudes of multilingualism (i.e., our participants may have already been interested in multilingualism and thus more likely to participate). Future research may benefit from careful sampling procedures to recruit those with less accepting attitudes toward multilingualism to fully assess the impacts of the PD. Lastly, this study did not measure changes in BCBAs’ practices; future research should measure the impacts of training at the provider and child levels in collaboration with family members.

## Conclusion

Bilingual autistic adults have reported that they value their ability to maintain or learn new languages (Digard et al., 2020, 2022; Nolte et al., 2021), and caregivers of autistic children have reported their desires to maintain their heritage languages with their children, but often struggle to do so for reasons such as the shortage of professionals with the knowledge and training to create learning environments that are supportive of children learning or maintaining their heritage languages (Durán et al., 2022; Fowler et al., 2019; Papoudi et al., 2021). This study found that a tailored PD could improve behavior analysts’ knowledge and self-efficacy toward supporting multilingual autistic children, their families, and the professionals who support them. More research and professional development opportunities are needed to improve behavior analysts’ and other autism providers’ practices to increase autistic children’s ability to maintain their heritage languages and learn new ones.

## Electronic supplementary material

Below is the link to the electronic supplementary material.

Supplementary Material 1

## Footnotes

1 Dual language education promotes multilingualism and literacy, academic achievement in multiple languages, and sociocultural competence (Center for Applied Linguistics, 2022).
