# Correction: Professional Development in Autism and Multilingualism for Behavior Analysts: A Randomized Waitlist Control Trial

**DOI:** 10.1007/s10803-025-06785-0

**Published:** 2025-03-13

**Authors:** Melanie R Martin Loya, Hedda Meadan, Xun Yan

**Affiliations:** 1 University of California Davis, MIND Institute, Sacramento, USA; 2 University of North Carolina at Charlotte, Charlotte, USA; 3 University of Illinois Urbana Champaign, Urbana, USA

**Journal of Autism and Developmental Disorders**

10.1007/s10803-025-06730-1

In this article, the title was incorrectly published as ‘Professional Development in Autism and Multilingualism for Behavior Analysts:’ but should have been ‘Professional Development in Autism and Multilingualism for Behavior Analysts: A Randomized Waitlist Control Trial’.

In the ‘Introduction’ section second paragraph, the following sentence was missing after the citation Zhou et al., 2019 ‘Researchers who employed single case methodology to examine multilingualism in children with autism or other developmental disabilities reported mixed results. In preference assessments, some children strongly preferred their families’ heritage language over English (e.g., Kunze et al., 2019)’

Incorrect Paragraph:

Research on the impacts of multilingualism on autistic children is nascent, but there is some evidence that growing up multilingual may benefit autistic children in executive functioning (Montgomery et al., 2021; Ratto et al., 2020, 2022; Sharaan et al., 2022) and social and language skills (Beauchamp et al., 2020; Siyambalapitiya et al., 2022; Zhou et al., 2019), while others indicated equal preference across languages (e.g., Padilla Dalmau et al., 2011). In addition, children sometimes displayed higher rates of challenging behaviors when presented with tasks in one language over another (e.g., Rispoli et al., 2011), and other children displayed minimal to no differentiation across language conditions when presented with tasks (e.g., Neely et al., 2019). Lastly, some learners engaged in higher rates of correct responses to academic and language tasks in their heritage language conditions (e.g., Lang et al., 2011), while others had improved responses in English-only conditions (e.g., León & Rosales, 2018). Varied results from the single-case studies highlight the importance of individualized assessment and intervention practices for multilingual autistic children and demonstrate the important role of the professionals who guide educational and clinical treatment plans for autistic children.

Corrected Paragraph:

Research on the impacts of multilingualism on autistic children is nascent, but there is some evidence that growing up multilingual may benefit autistic children in executive functioning (Montgomery et al., 2021; Ratto et al., 2020, 2022; Sharaan et al., 2022) and social and language skills (Beauchamp et al., 2020; Siyambalapitiya et al., 2022; Zhou et al., 2019). Researchers who employed single case methodology to examine multilingualism in children with autism or other developmental disabilities reported mixed results. In preference assessments, some children strongly preferred their families’ heritage language over English (e.g., Kunze et al., 2019), while others indicated equal preference across languages (e.g., Padilla Dalmau et al., 2011). In addition, children sometimes displayed higher rates of challenging behaviors when presented with tasks in one language over another (e.g., Rispoli et al., 2011), and other children displayed minimal to no differentiation across language conditions when presented with tasks (e.g., Neely et al., 2019). Lastly, some learners engaged in higher rates of correct responses to academic and language tasks in their heritage language conditions (e.g., Lang et al., 2011), while others had improved responses in English-only conditions (e.g., León & Rosales, 2018). Varied results from the single-case studies highlight the importance of individualized assessment and intervention practices for multilingual autistic children and demonstrate the important role of the professionals who guide educational and clinical treatment plans for autistic children.

The reference for the citation Chung et. 2022 was missing and should have read as ‘Chung, M. Y., Lee, J. D., Meadan, H., Sands, M. M., & Haidar, B. S. (2022). Building professionals’ capacity: The cascading coaching model. *Journal of Positive Behavior Interventions, 24*(4), 313–324. 10.1177/10983007211039295.

The original article has been corrected.

