# Supplementary material for: Professional Development in Autism and Multilingualism for Behavior Analysts: A Randomized Waitlist Control Trial
Source: J Autism Dev Disord. 2025 Feb 14;56(7):2625–37. doi: 10.1007/s10803-025-06730-1 (PMC13346295; doi:10.1007/s10803-025-06730-1)
Supplement: Supplementary file 1 — Supplementary Material 1 [file 10803_2025_6730_MOESM1_ESM.pdf]

Professional Development in Autism and Multilingualism for Behavior Analysts:

A Randomized Waitlist Control Trial - Supplemental Information

Melanie R Martin Loya, Hedda Meadan, Xun Yan

Table of Contents

Table: Study Design....2
Figure A....3
Figure B....4
Figure C....5
Screening and Demographic Form....6
Professional Development Training....14
Knowledge Assessment....25
General Self-Efficacy Scale....30
Language Attitudes of Teachers Scale....36
Social Validity Questionnaire....40

Table: Study Design

Randomized Waitlist Control Design

**Group**	**Time 1**<br>10 days	**Intervention**<br>10 days	**Time 2**<br>10 days	**Intervention**<br>10 days
**Intervention Group**	Pre-measures	X	Post-measures; Social validity questionnaire	Finished
**Waitlist Control Group**	Pre-measures	Waitlist	Post-measures	X<br>+ Social validity questionnaire

Figure A

Interaction Effect for Knowledge Assessment

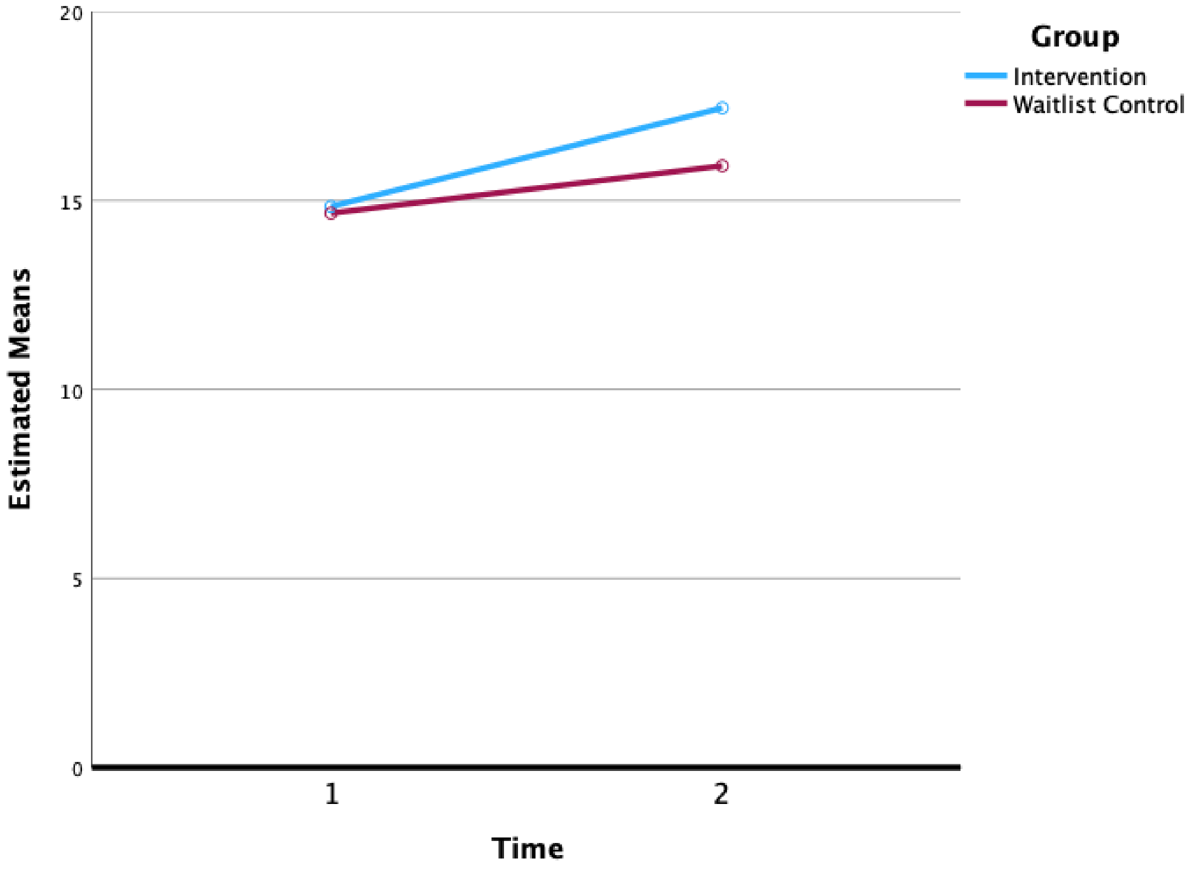

Note: The Y axis depicts the possible range of composite scores for the knowledge assessment, where 0=no correct answers and 19=all correct answers.

Fig. A depicts an interaction plot of time x group for the knowledge assessment. The X-axis has Time 1 and 2, and the Y-axis has "Estimated Means." Two lines of different colors depict intervention and waitlist groups. For Time 1, the intervention and waitlist group pre-scores are nearly identical, with overlapping points on the graph. For Time 2, both the intervention and waitlist groups increased the estimated means, but the intervention group has a steeper incline than the waitlist group and has a higher estimated mean.

Figure B

Interaction Effect for Self-Efficacy Scale

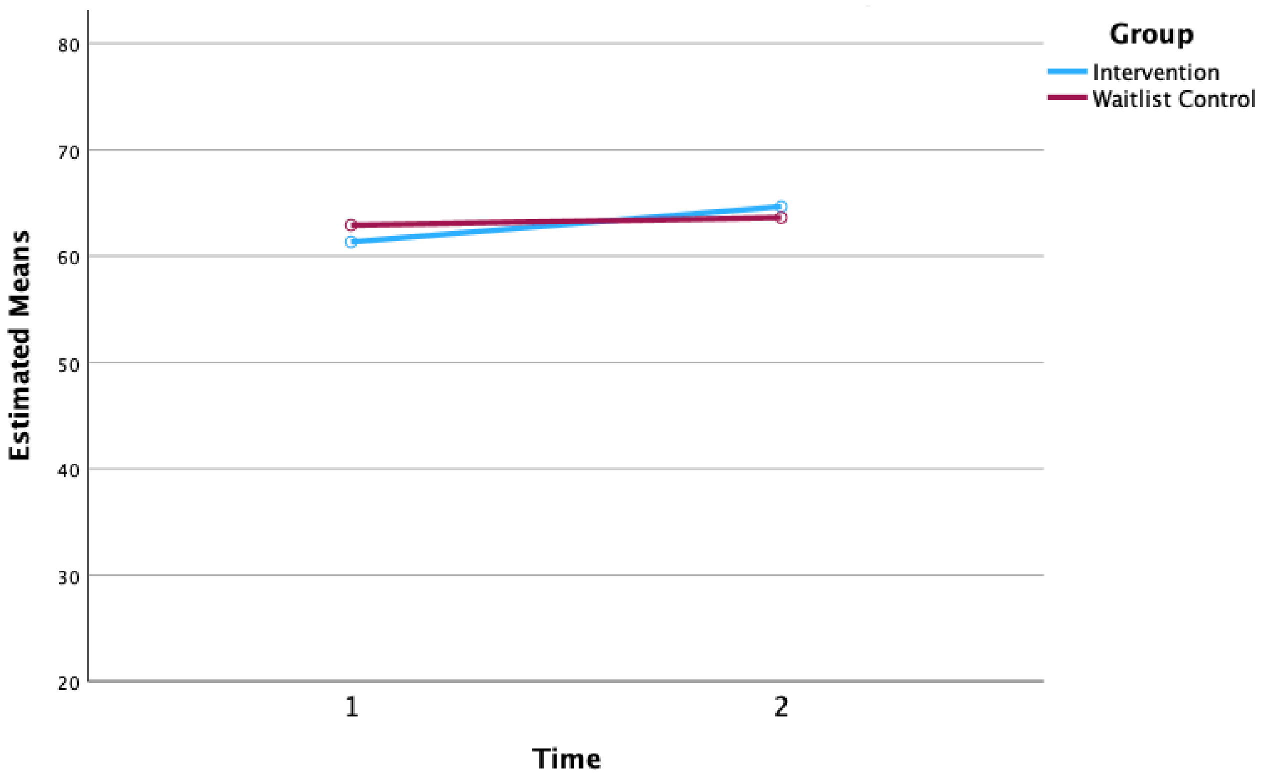

Note: The Y axis depicts the possible range of composite scores for the self-efficacy scale, where 20=low reported self-efficacy and 80=high reported self-efficacy.

Fig. B depicts an interaction plot of time x group for the self-efficacy assessment. The X-axis has Time 1 and 2, and the Y-axis has "Estimated Means." Two lines of different colors depict intervention and waitlist groups. For Time 1, the intervention and waitlist group pre-scores are nearly identical, with the intervention group having slightly lower estimated means. For Time 2, the waitlist groups had a flat line, and the intervention group had a slightly steeper incline than the waitlist group, resulting in a higher estimated mean in the intervention group.

Figure C

Interaction Effect for Attitude Scale

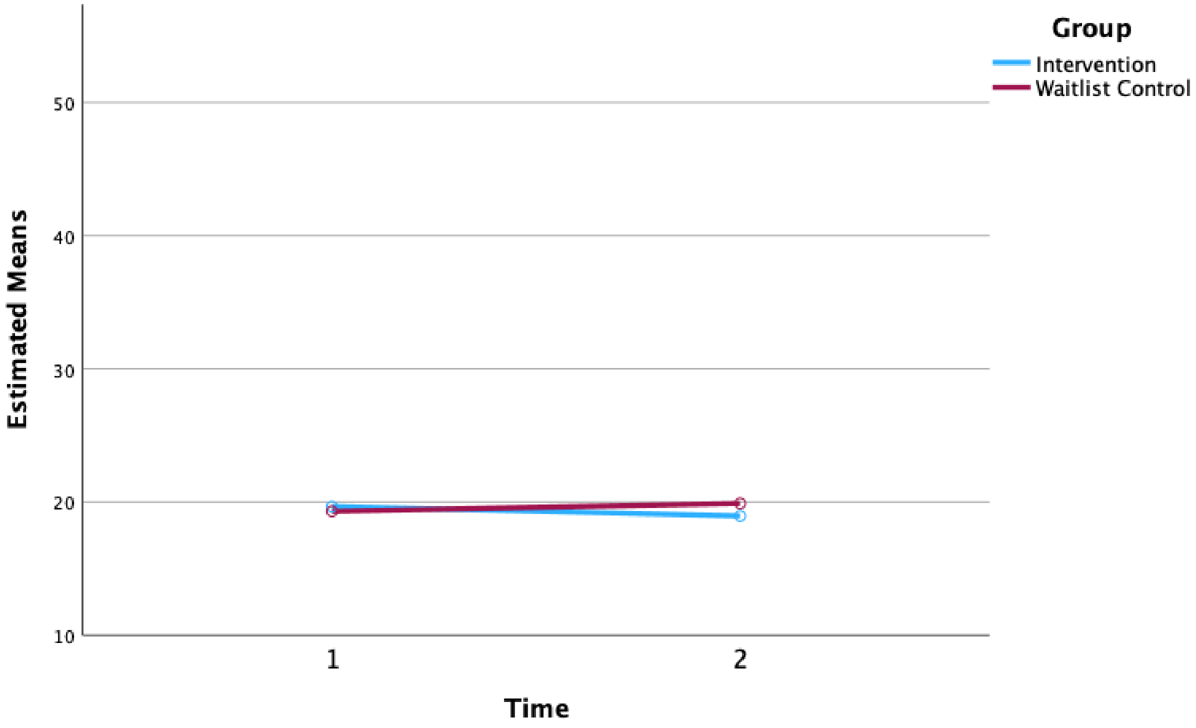

Note: The Y axis depicts the possible range of composite scores for the attitude scale, where 11=most favorable attitudes toward bilingualism and 55=least favorable attitudes toward bilingualism.

Fig. C depicts an interaction plot of time x group for the self-efficacy assessment. The X-axis has Time 1 and 2, and the Y axis has "Estimated Means." Two lines of different colors depict intervention and waitlist groups. For Time 1, the intervention and waitlist group pre-scores are nearly identical. For Time 2, both waitlist and intervention groups have a flat line, resulting in no significant changes in either group between Time 1 and Time 2's estimated mean.

Screening and Demographic Form

Please answer these questions to determine your eligibility to participate in the bilingual ABA professional development training module.

To avoid bots, the researcher will verify **ALL** participants before sending a link to participate in this study.

Please have your BCBA number handy!

Access to the CEU module will begin in a few weeks. Please keep an eye on your email to receive an invitation before contacting the researcher. Thank you for your patience and interest in this study. For any questions please reach out to Melanie**.**

Estimated time to complete this form is **less than 5 minutes.**

All questions with an asterisk * are required.

Email*: _______________

Screening Form - Highlights indicate required answer to participate

First and last name:* _______________

Are you currently certified as a Behavior Analyst (BCBA or BCBA-D) in good standing*?
*
*To be considered in good standing, you should not have any disciplinary actions associated with your certification, as defined by the BACB.
-Yes
-No

Please provide your certification number for verification. *
Please ensure you enter the correct number.

____

Have you been certified as a BCBA for more than one year?*
-Yes
-No

Have you completed the 8-hour Supervisor training and are eligible to supervise RBTs or those seeking BCBA certification?*
-Yes

-No

Are the primary recipients of your work children diagnosed with autism and their families?*
-Yes
-No

Do you currently or have you ever provided supervision or mentorship to RBTs or BCBAs?*
-Yes
-No

Are you over age 18, fluent in English, and do you live and work in the United States?*
-Yes
-No

Are you willing to participate in a 1-hour asynchronous online training and pre- and post-questionnaires totaling up to two hours and 20 minutes? *
-Yes
-No

Demographic Questionnaire

Start of Block: Demographic

Please indicate your current level of certification

If you have earned a relevant doctoral degree but do not have the doctoral designation, please select doctoral.

o BCBA - Masters level
o BCBA - Doctoral level

Age in years

Please use whole numbers only

____

Which best describes you?

o Female
o Male
o Transgender
o Non-binary
o Prefer not to share
o Other ____

Which best describes you? Select all that apply

▢ American Indian/Native American or Alaska Native
▢ Asian
▢ Black or African American
▢ Hispanic or Latino
▢ Middle Eastern or North African
▢ Native Hawaiian or Pacific Islander
▢ White or European American
▢ Other ____

Language(s) you have at least professional working proficiency:

Professional working proficiency is defined as the ability to speak the language with sufficient structural accuracy and vocabulary to participate effectively in most conversations on practical, social, and professional topics.

▢ English
▢ American Sign Language
▢ Arabic
▢ Chinese - Mandarin
▢ Chinese - Cantonese
▢ French
▢ Korean
▢ Russian
▢ Spanish
▢ Tagalog
▢ Vietnamese
▢ Other ____

What generation are you in the U.S.?

o First generation - I was born outside the continental U.S.
o Second generation - My parent(s) were born outside the U.S., and I was born in the continental U.S.
o Third generation - My grandparent(s) were born outside the U.S., and I and my parents were born in the continental U.S.
o Four or more generations in the U.S. or Unknown
o Prefer not to share

Number years of experience in ABA, including behavior technician or Registered Behavior Technician level, if applicable

Please use whole numbers only

____

In which state do you currently practice ABA?

If you practice in multiple states, please choose the state where you predominantly practice.

▼ Alabama ... Wyoming

Primary locations where your (or your supervisees') ABA services are provided:

Select all that apply

- ▢ Clients' homes
- ▢ Schools - Early Childhood
- ▢ Schools - K - 12
- ▢ Clinic
- ▢ Other ____

What is the age range of the children receiving services under your care or supervision?

Select all that apply

▢ Birth - 2
▢ 3 - 5 Preschool
▢ 6 - 8 Lower middle childhood
▢ 9 - 11 Upper middle childhood
▢ 12 - 14 Young teen
▢ 15 - 17 Teenagers
▢ 17+

How would you **best** describe your current role?

o 1. I primarily work 1:1 with clients (i.e., I provide direct support to children or their caregivers and I do not supervise RBTs or behavior technicians)
o 2. I primarily provide **case management and supervision of RBTs, behavior technicians, or BCaBAs** (e.g., I might travel to individual client's homes and/or schools to provide support or I stay within one clinical setting; may also involve activities from Option 1)
o 3. Director - Local: I primarily provide **higher level clinical and administrative support within a single location** (e.g., one school or clinic; may also involve activities from Options 1 and 2)
o 4. Director - Regional and beyond: I primarily provide **higher level clinical and administrative support across various distinct locations** (e.g., across various cities; may also involve activities from Options 1 and 2)
o 5. Higher education or professional supervision: I primarily **teach or provide structured supervision** to students accruing BACB fieldwork/practicum hours.
o Other ____

Have you ever supervised BCBAs or behavior technicians who were providing services in a language other than English?

o No
o Yes

Have you ever supported children or families who primarily communicated in a language other than English?

- o No
- o Yes

Have you received any training or mentorship specific to supporting children on the autism spectrum from heritage language (i.e., non-English) speaking homes?

- o Yes
- o No

Please select what training or mentorship you received specific to supporting bilingual children

- ▢ Direct supervision or mentorship from a bilingual individual related to providing bilingual care
- ▢ Attended a CEU event online or in person
- ▢ University coursework related to supporting bilingual children or staff
- ▢ Other ____

Have you received any training or mentorship specific to supporting bilingual staff who work with children on the autism spectrum from heritage language (i.e., non-English) speaking homes?

- o Yes
- o No

Please select what training or mentorship you received specific to supporting bilingual staff

- ▢ Direct supervision or mentorship from a bilingual individual related to providing bilingual care
- ▢ Attended a CEU event online or in person
- ▢ University coursework related to supporting bilingual children or staff
- ▢ Other ____

What roles or identities do you hold related to the autism/autistic community?

- ▢ I identify as autistic/someone on the autism spectrum.
- ▢ I am a parent or caregiver of someone autistic/on the autism spectrum.
- ▢ I am a sibling of someone autistic/on the autism spectrum.
- ▢ I am the child of someone autistic/on the autism spectrum.
- ▢ I am a close family member of someone autistic/on the autism spectrum. (e.g., grandparent, aunt/uncle, cousin).
- ▢ I am a close friend to someone autistic/on the autism spectrum.
- ▢ I am in a romantic partnership with someone autistic/on the autism spectrum.
- ▢ I have no additional connections to autism outside of my profession.
- ▢ I prefer not to share.
- ▢ Other ____

End of Block: Demographic

Professional Development Training

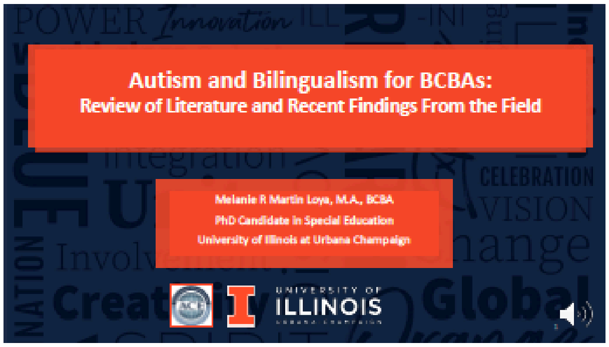

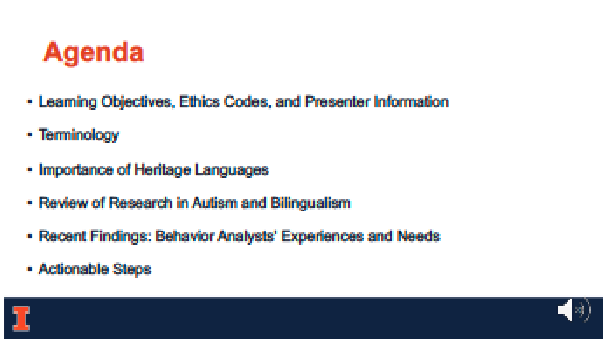

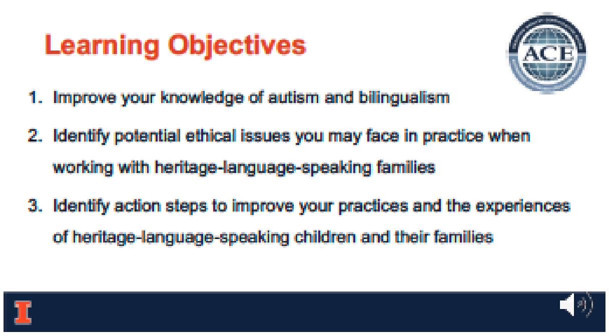

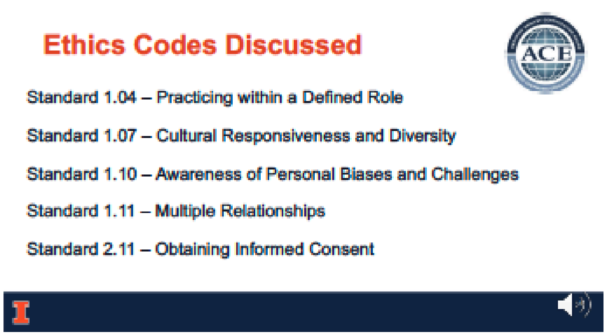

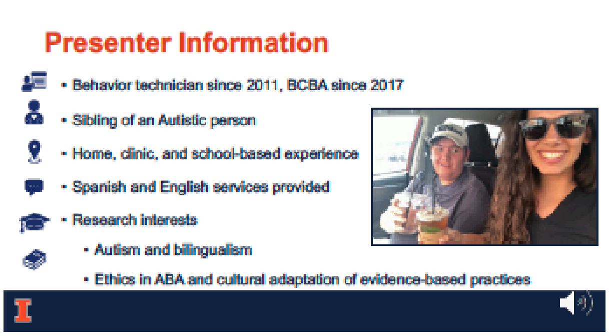

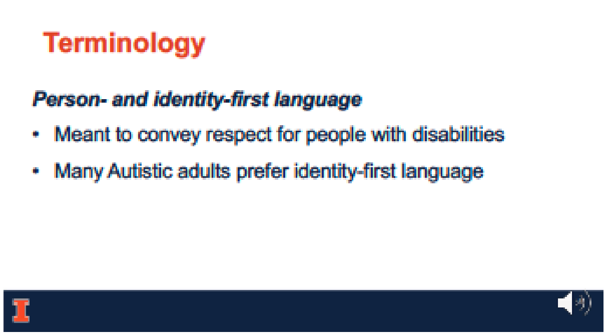

Terminology

Heritage language

- Home language that is different from the majority language in a society (i.e., any language other than English in the U.S.)
- Heritage-language-speakers often labeled *English Learners or Dual Language Learners*

Terminology

Bilingual

- Anyone who speaks or *is regularly exposed to* more than one language
- Bilingualism is a *spectrum* of skills across domains (e.g., reading, writing, speaking)

Terminology

Dually Identified

1. Having a disability (e.g., autism)
2. Bilingual

Often are also members of historically marginalized groups (e.g., immigrants or children of immigrants)

Terminology

Interpreter	***Translator***
• Mediates language in-vivo<br>"Please direct questions to the caregivers and the *interpreter* will help them understand throughout this IEP meeting."	• Mediates language with writing<br>"The *translator* will have the assessments *translated* into Spanish and ready to send to parents by Monday."

Terminology

Interpreters and Translators…

… are skilled professionals. Bilingualism is a prerequisite to be an interpreter or a translator, but **not all bilingual individuals are qualified to be interpreters or translators in a professional capacity.**

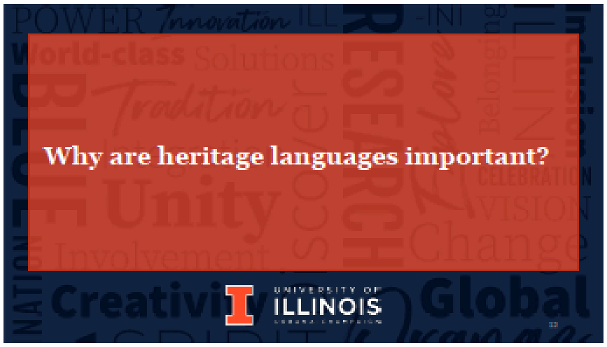

Importance of Heritage Languages

In those States in which ethnic, religious or linguistic minorities or persons of indigenous origin exist, a child belonging to such a minority or who is indigenous shall not be denied the right, in community with other members of his or her group, to enjoy his or her own culture, to profess and practice his or her own religion, or to use his or her own language. (UNCRC, 1989)

Importance of Heritage Languages

Heritage-language-speaking families of autistic children report:

- Desire to maintain their language (e.g., Papoudi et al., 2020)
- Being discouraged from maintaining their heritage language with their children (e.g., Drysdale et al., 2015)
- Difficulties finding bilingual providers (e.g., Siyambalapitiya et al., 2021)

Importance of Heritage Languages

Autistic adults have self-reported positive impacts of being bilingual including improved:

- Social connections and relationships
- Communication skills
- Access to hobbies
- Cultural insights

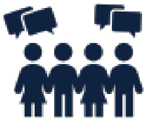

(Digard et al., 2020; 2022; Nolte et al., 2021)

So, heritage languages are important – now what?

"Continually increase [our] knowledge and skills related to cultural responsiveness and service delivery to diverse groups."

-Standard 1.07 - The Ethics Code for Behavior Analysts (2020)

So, heritage languages are important – now what?

Improve our understanding of the experiences of dually identified children, their families, and those who support them

- e.g., Educational experiences and rights

Understand research related to dually identified children

- e.g., What's known about their academic/language outcomes?

Educational Experiences

Dually identified children and their families report:

- Strong desire for their children to succeed in school (e.g., Ansari et al., 2018; Yosso, 2005)
- Less knowledge about special education and more negative experiences (e.g., discrimination; Burke et al., 2020; 2021)

Individuals with Disabilities Education Act (IDEA)

IDEA is a federal law that entitles children with disabilities access to a free and appropriate public education and ensures they receive special education services.

Legal Rights of Dually Identified Children

*School districts **must** provide [dually identified students] with **both** the language assistance and disability-related services to which they are entitled under Federal law.*

U.S. Department of Education (2015)

What do we know about the impacts of bilingualism on autistic child outcomes?

UNIVERSITY OF ILLINOIS

Review of Research: Single-Case

Child preference assessment of language	Impact of language on treatment efficacy or outcomes
1.Aguilar et al. (2017)<br>2.Aguilar et al. (2016)<br>3.Clay et al. (2020)<br>4.Kunze et al. (2019)<br>5.Padilla Dalmau et al. (2011)*	1.Banerjee et al. (2022)<br>2.Lang et al. (2011)<br>3.León & Rosales (2018)<br>4.Lim & Charlop (2018)<br>5.Neely et al. (2020)<br>6.Neely et al. (2022)<br>7.Rispoli et al. (2011)<br>8.Padilla Dalmau et al. (2011)*

Review of Research: Single-Case

Child preference assessment of language	Impact of language on treatment efficacy or outcomes
• Preferences varied!<br>• Some prefer English<br>• Some prefer heritage language<br>• Some had minimal preference<br>• Always measure child language preferences	• Impacts varied!<br>• Many benefited from their heritage language<br>• History of reinforcement across languages is important<br>• Incorporate heritage languages into treatment and measuring impacts across languages

Review of Research: Group Design

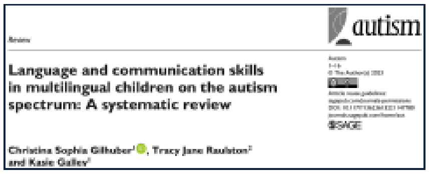
Review

autism

Language and communication skills in multilingual children on the autism spectrum: A systematic review

Christina Sophia Gilhuber[1], Tracy Jane Raulston[2] and Kasie Galley[1]

SAGE

Review of Research: Group Design

- No evidence of negative effects, some evidence of positive effects.
- Language and communication skills were similar between monolingual and bilingual autistic children
- Results do not support the common misconception that autistic children learn best in one language
- Study participants **not** representative of the autistic community
  - Children with co-occurring conditions (e.g., intellectual disability or complex communication needs) were often excluded

Review of Research – General Implications

- Reinforcement history, functions of behavior, and preferences vary!
- Potential advantages of bilingualism
- High social validity for families and autistic adults and positive impacts on quality of life

What do we know about behavior analysts' experiences supporting dually identified children?

UNIVERSITY OF ILLINOIS URBANA-CHAMPAIGN

Recent Findings: Experiences of BCBAs

Martin Loya & Meadan – *2022 study*:

- One-to-one interviews with 16 bilingual behavior analysts in the U.S.

Martin Loya & Meadan – *current study*:

- Focus groups and one-to-one interviews with 16 monolingual and bilingual behavior analysts to gain a more complete picture and understanding of experiences, challenges, and needs – *with an added focus on supporting bilingual ABA staff*

Findings across studies

- Behavior analysts' experiences
  - Positives
  - Challenges
- Behavior analysts' needs
  - Translated Resources
  - Access to Interpreters
  - Access to Training

Findings – Positive Experiences

This sub-theme has additional categories:

1. Improved connection and rapport with families and colleagues
2. Empathy and understanding toward families
3. Strongly valuing bilingualism

Findings – Positive Experiences

Improved connection and rapport with families and colleagues.

Bilingual behavior analysts described how sharing the same language with families improved the:

- Ease, speed, and quality

… Of building meaningful connections with the families they served.

"I am able to establish that rapport real fast with Spanish speaking families."

Findings – Positive Experiences

High levels of empathy and understanding toward families.

Bilingual behavior analysts shared how:

- Language and culture are often intertwined
- Empathy and understanding toward their families' intersecting identities is important

"Understanding the lack of not knowing the language, especially when they're trying to get services for their kids [is so important]."

Ethics Codes – Positive Demonstrations

Standard 1.07 – Cultural Responsiveness and Diversity

"Whether you're Hispanic or not, you do have to understand that not everybody shares your background… you have to make efforts to really understand where they're coming from."

Ethics Codes – Positive Demonstrations

Standard 1.10 – Awareness of Personal Biases and Challenges

"I'm Mexican American, and whenever I would go back to Mexico and visit my cousins…. They would single me out… I remember experiencing that. I don't want [my Chinese student] to have to feel that way [when they visit China] because here I am stepping over them or their culture… I was always trying to be overly cautious in doing that, and I was honest with [my clients] like, I just really want to make sure that [they] feel comfortable."

Findings – Positive Experiences

Strongly valuing bilingualism.

Bilingual behavior analysts shared experiences that demonstrated how highly they value bilingualism because it:

- Improves family buy-in
- Promotes positive clinical outcomes for children

"We're there to support [heritage-language-speaking clients] too, not just English-speaking clients."

Findings – Positive Experiences

Positive clinical outcomes:

- Reduction in behaviors such as self-injury
- Increases in behaviors such as communication and self-advocacy skills

… after switching from English-only care to heritage-language care

Findings – Challenging Experiences

This sub-theme has additional categories:

1. Challenges with translation and interpretation
2. Discrimination by families and colleagues
3. Overworked, underappreciated, and blurred lines at work

Findings – Challenging Experiences

Challenges with translation and interpretation.

Bilingual behavior analysts described challenges related to:

- Technical jargon
- Professional training and graduate school being entirely in English

"I went from English… to Spanish, with no training or preparation of the ABA words."

Findings – Challenging Experiences

Experiences of Discrimination.

Bilingual behavior analysts described experiencing discrimination from:

- English-only speaking families
- English-only speaking colleagues

"…We were 'the spicy clinic'…"

Findings – Challenging Experiences

Overworked, Underappreciated, and Blurred Lines.

Behavior analysts described additional challenges related to:

- Burnout and often being the only bilingual BCBA at work
- Having additional uncompensated burdens at work
- Ethical challenges

"… I've seen the burnout in bilingual BCBAs…"

Ethics Codes – Challenging Implications

Standard 1.04 – Practicing Outside a Defined Role

"I stop supervising and I become a translator."

Standard 1.11 – Multiple Relationships

"I feel like in Spanish speaking homes, sometimes it can be so casual when parents bring up things that are outside of the treatment plan… that happens more in my bilingual homes than my non-bilingual homes."

Ethics Codes – Challenging Implications

Standard 2.11 – Obtaining Informed Consent

"How many more hours did I do because I had to sit and translate the assessment so that the family understood, and I can appropriately get consent for that document? … I think there's a lot of that stuff that gets missed because we're bilingual."

Findings – Behavior Analysts' Needs

Informed by 2022 study and reaffirmed in 2023 focus groups

1. Translated Materials and Resources
2. Access to Interpreters
3. Increased Recruitment of Bilingual Providers
4. Additional Training for Families and Providers

"I'm one person. I can't be everywhere."

Discussion – Focus Groups + Interviews

Major takeaways:

- Behavior analysts find meaning and joy providing bilingual support
- Reports of benefits due to bilingualism
- Bilingual and monolingual BCBAs report similar needs
- Specific challenges related to structural and interpersonal barriers – implicating several Ethics Codes (BACB, 2020)

Action Steps – Research and Higher Ed

- Incorporate literature on language and bilingualism into behavior analytic coursework and supervision
- Explore additional training or licensure options for bilingual behavior analysts, ASHA may serve as an example
- Additional research in this area is sorely needed
  - Researchers with lived and/or professional experience in providing bilingual service are encouraged to lead these efforts

Action Steps – Organization

- Leadership are encouraged to learn about bilingualism
- Examine evaluation practices to ensure fairness and equity
- Invest in bilingual staff, translation, and interpretation services
- Network with other local and regional centers to share language resources

Action Steps – Organization

- Leadership are encouraged to learn about bilingualism
- Examine evaluation practices to ensure fairness and equity
- Invest in bilingual staff, translation, and interpretation services
- Network with other local and regional centers to share language resources

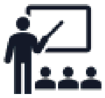

Action Steps – Organization

- Leadership are encouraged to learn about bilingualism
- Examine evaluation practices to ensure fairness and equity
- Invest in bilingual staff, translation, and interpretation services
- Network with other local and regional centers to share language resources

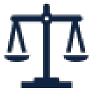

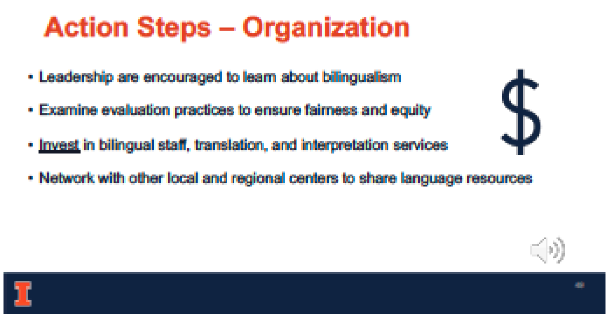
Action Steps – Organization
• Leadership are encouraged to learn about bilingualism
• Examine evaluation practices to ensure fairness and equity
• Invest in bilingual staff, translation, and interpretation services
• Network with other local and regional centers to share language resources

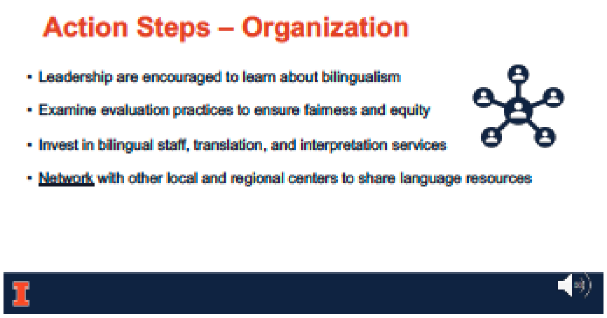
Action Steps – Organization
• Leadership are encouraged to learn about bilingualism
• Examine evaluation practices to ensure fairness and equity
• Invest in bilingual staff, translation, and interpretation services
• Network with other local and regional centers to share language resources

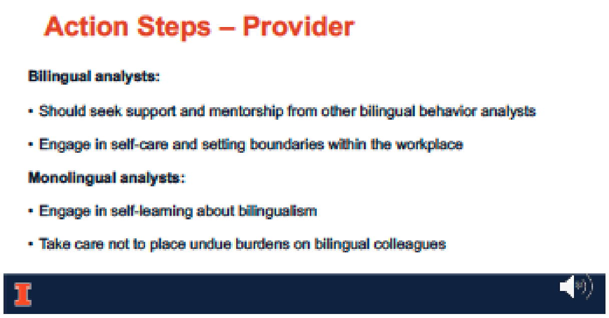
Action Steps – Provider
Bilingual analysts:
• Should seek support and mentorship from other bilingual behavior analysts
• Engage in self-care and setting boundaries within the workplace
Monolingual analysts:
• Engage in self-learning about bilingualism
• Take care not to place undue burdens on bilingual colleagues

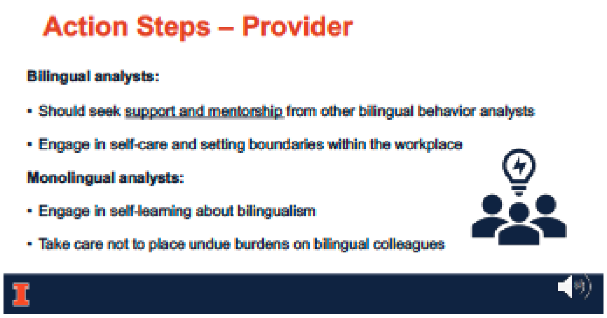
Action Steps – Provider
Bilingual analysts:
• Should seek support and mentorship from other bilingual behavior analysts
• Engage in self-care and setting boundaries within the workplace
Monolingual analysts:
• Engage in self-learning about bilingualism
• Take care not to place undue burdens on bilingual colleagues

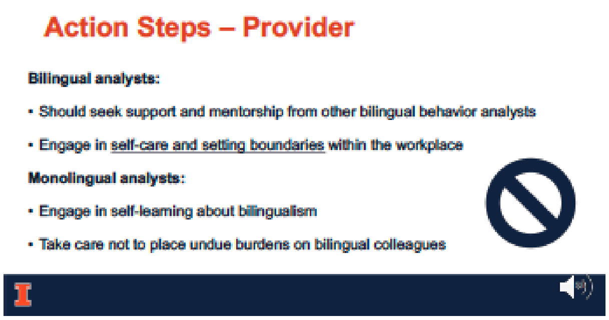
Action Steps – Provider
Bilingual analysts:
• Should seek support and mentorship from other bilingual behavior analysts
• Engage in self-care and setting boundaries within the workplace
Monolingual analysts:
• Engage in self-learning about bilingualism
• Take care not to place undue burdens on bilingual colleagues

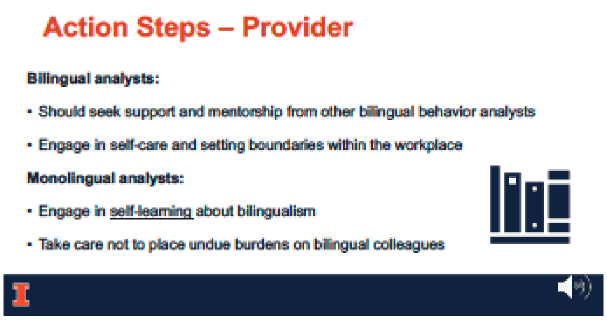
Action Steps – Provider
Bilingual analysts:
• Should seek support and mentorship from other bilingual behavior analysts
• Engage in self-care and setting boundaries within the workplace
Monolingual analysts:
• Engage in self-learning about bilingualism
• Take care not to place undue burdens on bilingual colleagues

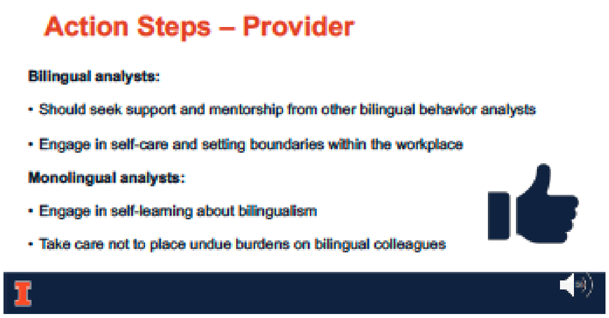
Action Steps – Provider
Bilingual analysts:
• Should seek support and mentorship from other bilingual behavior analysts
• Engage in self-care and setting boundaries within the workplace
Monolingual analysts:
• Engage in self-learning about bilingualism
• Take care not to place undue burdens on bilingual colleagues

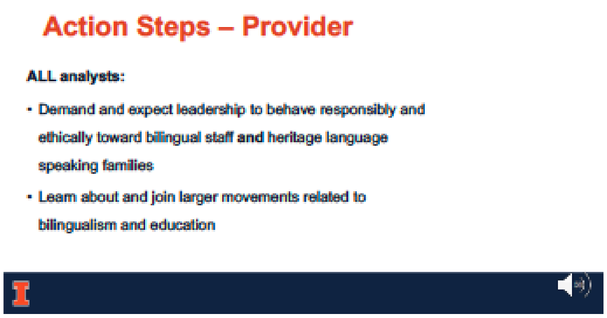
Action Steps – Provider
ALL analysts:
• Demand and expect leadership to behave responsibly and ethically toward bilingual staff and heritage language speaking families
• Learn about and join larger movements related to bilingualism and education

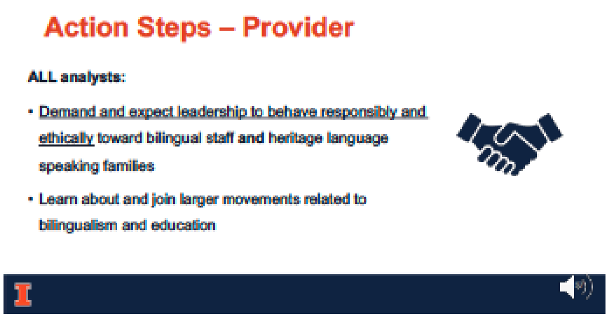
Action Steps – Provider
ALL analysts:
• Demand and expect leadership to behave responsibly and ethically toward bilingual staff and heritage language speaking families
• Learn about and join larger movements related to bilingualism and education

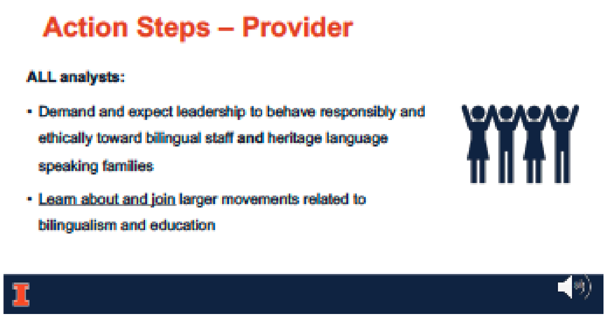
Action Steps – Provider
ALL analysts:
• Demand and expect leadership to behave responsibly and ethically toward bilingual staff and heritage language speaking families
• Learn about and join larger movements related to bilingualism and education

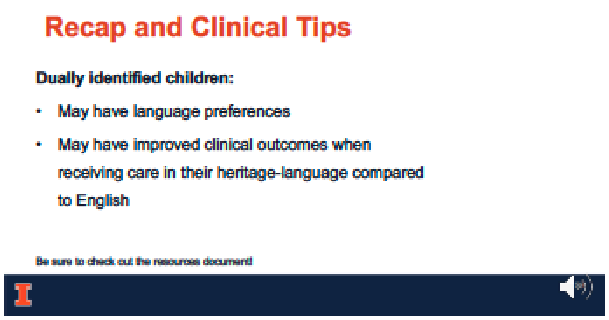
Recap and Clinical Tips
Dually identified children:
• May have language preferences
• May have improved clinical outcomes when receiving care in their heritage-language compared to English
Be sure to check out the resources document!

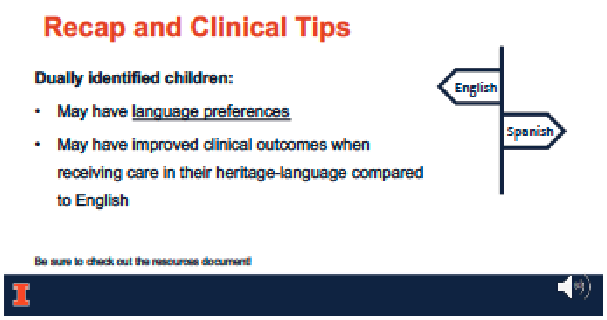
Recap and Clinical Tips
Dually identified children:
• May have language preferences
• May have improved clinical outcomes when receiving care in their heritage-language compared to English
English
Spanish
Be sure to check out the resources document!

Recap and Clinical Tips

Dually identified children:

- May have language preferences
- May have improved clinical outcomes when receiving care in their heritage-language compared to English

Be sure to check out the resources document!

Recap and Clinical Tips

BEFORE intervention (during intake):

Learn about children's language environments

- e.g., school, home, community

Assess family preferences and values related to language

- e.g., How comfortable are family members across languages? Will the child lose or gain access to important communication partners?

Be sure to check out the resources document!

Recap and Clinical Tips

BEFORE intervention (during intake):

Learn about children's language environments

- e.g., school, home, community

Assess family preferences and values related to language

- e.g., How comfortable are family members across languages? Will the child lose or gain access to important communication partners?

Be sure to check out the resources document!

Recap and Clinical Tips

BEFORE intervention (during intake):

Learn about children's language environments

- e.g., school, home, community

Assess family preferences and values related to language

- e.g., How comfortable are family members across languages? Will the child lose or gain access to important communication partners?

Be sure to check out the resources document!

Recap and Clinical Tips

DURING intervention, measure and analyze:

Child language preferences

- Periodic probes, preference assessments, etc.

Impacts of language of instruction

- Behavioral increases (e.g., verbal behavior)
- Behavior decreases (e.g., self-injurious behavior)

Be sure to check out the resources document!

Recap and Clinical Tips

Remember!

We have the skills to assess and monitor behavior. Use your skills to determine how/if language of instruction impacts child preferences and behavioral increases or decreases.

Make clinical decisions accordingly, in collaboration with caregivers, and the knowledge that bilingualism has no known negative impacts – only positive!

Just don't forget to include language!

Knowledge Assessment

Please do not attempt to google or otherwise search for the answers. This is a pre-assessment to determine your knowledge prior to watching the training video. Wrong answers will not impact your ability to earn the CEU. Honesty is highly appreciated. Thank you.

Children on the autism spectrum are known to learn best in one language.

- o False
- o True

Which of the following are true:

Select all that apply

- ▢ A translator is someone who works with written documents
- ▢ Translators and interpreters are interchangeable.
- ▢ All bilingual people can work as interpreters or translators.
- ▢ An interpreter is someone who mediates between languages in-vivo (e.g., orally or through signing).

Bilingualism is considered a spectrum. Some bilingual individuals may have skills across domains in both languages, and some bilingual individuals may be stronger in one domain compared to another (e.g., able to communicate orally with proficiency, but unable to translate documents or write in both languages with precision).

- o False
- o True

Heritage-language-speaking caregivers of autistic children have reported that:

- o Many professionals encourage them to maintain their heritage language with their children.
- o Many professionals discourage them from maintaining their heritage language with their children.
- o Maintaining their heritage language with their children in the U.S. in easy.
- o Maintaining their heritage language with their children in the U.S. is not a priority for them.

Heritage-language-speaking caregivers of autistic children have reported having less knowledge about special education and more negative experiences with the school system compared to English-speaking caregivers.

- o False
- o True

Fill in the blank:

Most bilingual behavior analysts in Martin Loya and Meadan have reported receiving [____] compensation for providing heritage language services.

- o additional monetary
- o no additional monetary

Fill in the blank:

Bilingual behavior analysts in Martin Loya and Meadan (2022) reported that sharing the same language [___] with the families they served.

- o made it difficult to connect
- o improved the ease and quality of their connections
- o did not impact their ability to connect
- o decreased the ease and quality of their connections

Fill in the blank:

Bilingual behavior analysts in Martin Loya and Meadan (2022) reported that many bilingual children on their caseloads experienced [____] upon receiving heritage-language care.

Select all that apply

- ▢ a reduction of behaviors such as self-injury
- ▢ increases in behaviors such as communication and self-advocacy skills
- ▢ no clinical impacts
- ▢ undesirable clinical impacts
- ▢ increases in behaviors such as self-injury
- ▢ a reduction of behaviors such as communication and self-advocacy skills

---

Fill in the blank:

Children dually identified with autism under IDEA and as “English learners” in the U.S. public school system are entitled to receive [1] special education [2] language support services.

- (1) both; (2) and
- (1) neither; (2) nor
- (1) only; (2) not
- (1) no; (2) and only

Bilingual behavior analysts in Martin Loya and Meadan (2022) reported:

- Feeling under appreciated for their efforts when supporting heritage-language-speaking families.
- Wanting more translated resources (e.g., assessments) to improve the quality of their work.
- Often being the only bilingual BCBA at their place of work.
- Experiencing discrimination from monolingual colleagues and clients.
- All of the above

In a systematic review of 22 studies on bilingualism in autistic children and its impact on children’s language and communication skills, Gilhuber, Raulston, and Galley (2023) reported:

- Participants in the included studies were a **good** representative sample of all autistic children.
- Participants in the included studies were **not a good** representative sample of all autistic children because children with co-occuring intellectual disability were often excluded from participating in research.
- Bilingual autistic children experience more negative impacts on their language and communication skills compared to monolingual autistic children.
- All bilingual autistic children experience significant positive impacts on their language and communication skills compared to monolingual autistic children.

In single case studies, dually identified children:

- o Always demonstrated strong language preferences
- o Tended to prefer their heritage language over English
- o Tended to prefer English over their heritage language
- o Had varied language preferences

Single case researchers recommended:

Select all that apply

- ▢ Considering history of reinforcement across languages
- ▢ Incorporating heritage languages into treatment
- ▢ Measuring the impact(s) of language of instruction
- ▢ Measuring child language preferences

Autistic adults have reported positive impacts of being bilingual including improved social connections, communication skills, access to hobbies, and cultural insights.

- o False
- o True

General Self-Efficacy Scale

adapted from Schwarzer & Jerusalem, 1995

Please note the following definitions when answering the questions:

Heritage-language: In the context of the U.S. this means any language other than English. *Example* - I have three heritage-language speaking families on my caseload. Two families speak Spanish and one speaks Vietnamese.

Direct-care staff: This includes anyone who works one-to-one with children and/or caregivers who are under your supervision or guidance. If you are in higher education, please consider relevant students/supervisees as direct-care staff. *Example* – I have five direct-care staff members, four are RBTs and one is a recently certified BCBA.

I can always manage to solve difficult problems [___] if I try hard enough.

	Not at all true	Hardly true	Moderately true	Exactly true
With heritage-language-speaking families	o	o	o	o
With direct-care staff who work with heritage-languag	o	o	o	o

e-speaking families	

If someone opposes me, I can find the means and ways to get what I want [___].

	Not at all true	Hardly true	Moderately true	Exactly true
With heritage-languag e-speaking families	o	o	o	o
With direct-care staff who work with heritage-languag e-speaking families	o	o	o	o

It is easy for me to stick to my aims and accomplish my goals [___].

	Not at all true	Hardly true	Moderately true	Exactly true
With heritage-languag	o	o	o	o

e-speaking families				
With direct-care staff who work with heritage-language-speaking families	o	o	o	o

I am confident that I could deal efficiently with unexpected events [___].

	Not at all true	Hardly true	Moderately true	Exactly true
With heritage-language-speaking families	o	o	o	o
With direct-care staff who work with heritage-language-speaking families	o	o	o	o

Thanks to my resourcefulness, I know how to handle unforeseen situations [___].

	Not at all true	Hardly true	Moderately true	Exactly true
With heritage-languag e-speaking families	o	o	o	o
With direct-care staff who work with heritage-languag e-speaking families	o	o	o	o

I can solve most problems if I invest the necessary effort [___].

	Not at all true	Hardly true	Moderately true	Exactly true
With heritage-languag e-speaking families	o	o	o	o
With direct-care staff who work with heritage-languag	o	o	o	o

e-speaking families				

I can remain calm when facing difficulties [___] because I rely on my coping abilities.

	Not at all true	Hardly true	Moderately true	Exactly true
With heritage-languag e-speaking families	o	o	o	o
With direct-care staff who work with heritage-languag e-speaking families	o	o	o	o

When I am confused with a problem [___], I can usually find several solutions.

	Not at all true	Hardly true	Moderately true	Exactly true
With heritage-languag	o	o	o	o

e-speaking families				
With direct-care staff who work with heritage-language-speaking families	o	o	o	o

If I am in trouble [___], I can usually think of a solution.

	Not at all true	Hardly true	Moderately true	Exactly true
With heritage-language-speaking families	o	o	o	o
With direct-care staff who work with heritage-language-speaking families	o	o	o	o

I can usually handle whatever comes my way [___].

	Not at all true	Hardly true	Moderately true	Exactly true
With heritage-language-speaking families	o	o	o	o
With direct-care staff who work with heritage-language-speaking families	o	o	o	o

End of Block: Self-Efficacy

Language Attitudes of Teachers Scale

(adapted from Byrnes & Kiger, 1994, Flores & Smith, 2009, and Cho et al., 2023)

To be considered American, one should communicate in English.

- o Strongly disagree
- o Disagree
- o Uncertain
- o Agree
- o Strongly agree

English should be the official language of the United States.

- o Strongly disagree
- o Disagree
- o Uncertain
- o Agree
- o Strongly agree

Local and state governments should require that all government or government-funded business (e.g., services for children with autism and their families) are conducted only in English.

- o Strongly disagree
- o Disagree
- o Uncertain
- o Agree
- o Strongly agree

Too much time and energy are now being placed on multiculturalism in education, allied health care (e.g., ABA), and society.

- o Strongly disagree
- o Disagree
- o Uncertain
- o Agree
- o Strongly agree

The rapid learning of English should be a priority for non-English-proficient or limited-English-proficient children with autism even if it means they lose the ability to communicate in their native/heritage language.

- o Strongly disagree
- o Disagree
- o Uncertain
- o Agree
- o Strongly agree

The government should spend additional money to provide better programs for children from underrepresented linguistic backgrounds in public schools and other human services (e.g., ABA).

- o Strongly disagree
- o Disagree
- o Uncertain
- o Agree
- o Strongly agree

Behavior analysts should modify their instruction for their students' cultural and linguistic needs.

- o Strongly disagree
- o Disagree
- o Uncertain
- o Agree
- o Strongly agree

Behavior analysts should be required to receive training to be prepared to meet the needs of children and families from underrepresented linguistic backgrounds.

- o Strongly disagree
- o Disagree
- o Uncertain
- o Agree
- o Strongly agree

It is unreasonable to expect a behavior analyst to teach a child or provide caregiver support to someone who does not communicate in English.

- o Strongly disagree
- o Disagree
- o Uncertain
- o Agree
- o Strongly agree

Most non- and limited-English-proficient children are not motivated to learn English.

- o Strongly disagree
- o Disagree
- o Uncertain
- o Agree
- o Strongly agree

Even when they do speak English, heritage-language-speaking caregivers don't participate in ABA-related activities as other caregivers do.

- o Strongly disagree
- o Disagree
- o Uncertain
- o Agree
- o Strongly agree

End of Block: Attitudes

Social Validity Questionnaire

Start of Block: Social Validity

Q1 Social Validity - Content and Goals

Q2 I am satisfied with the content covered in this online training.

- o Strongly disagree (1)
- o Somewhat disagree (2)
- o Neither agree nor disagree (3)
- o Somewhat agree (4)
- o Strongly agree (5)

Q3 I believe the content covered in this online training is important for advancing equity in ABA services.

- o Strongly disagree (1)
- o Somewhat disagree (2)
- o Neither agree nor disagree (3)
- o Somewhat agree (4)
- o Strongly agree (5)

Q4 I would recommend the training to other ABA practitioners.

- o Strongly disagree (1)
- o Somewhat disagree (2)
- o Neither agree nor disagree (3)
- o Somewhat agree (4)
- o Strongly agree (5)

Q5 The content covered in this online training used appropriate and inclusive language.

- o Strongly disagree (1)
- o Somewhat disagree (2)
- o Neither agree nor disagree (3)
- o Somewhat agree (4)
- o Strongly agree (5)

Q6 Would you like to share anything else about the acceptability of the training goals and content?

Optional

____

____

Q7 Social Validity - Procedures

Q8 I felt the length of the training was appropriate for the content being taught.

- o Strongly disagree (1)
- o Somewhat disagree (2)
- o Neither agree nor disagree (3)
- o Somewhat agree (4)
- o Strongly agree (5)

Q9 I found the online training video to be easy to watch.

- o Strongly disagree (1)
- o Somewhat disagree (2)
- o Neither agree nor disagree (3)
- o Somewhat agree (4)
- o Strongly agree (5)

Q10 I found the pop-up questions in the online training video to be easy to navigate.

- o Strongly disagree (1)
- o Somewhat disagree (2)
- o Neither agree nor disagree (3)
- o Somewhat agree (4)
- o Strongly agree (5)

Q11 Would you like to share anything else about the procedures of the training?

Optional

____

____

Q12 Social Validity - Outcomes

Q13 The information in the online training improved my understanding of autism and bilingualism.

- o Strongly disagree (1)
- o Somewhat disagree (2)
- o Neither agree nor disagree (3)
- o Somewhat agree (4)
- o Strongly agree (5)

Q14 The information in the online training improved my understanding of benefits, needs, and challenges commonly experienced by bilingual BCBAs.

- o Strongly disagree (1)
- o Somewhat disagree (2)
- o Neither agree nor disagree (3)
- o Somewhat agree (4)
- o Strongly agree (5)

Q15 I feel more prepared to support dually identified children and families as a result of this training.

- o Strongly disagree (1)
- o Somewhat disagree (2)
- o Neither agree nor disagree (3)
- o Somewhat agree (4)
- o Strongly agree (5)

Q16 As a result of this training, I plan to engage in at least one action to improve my or my organization's practices related to bilingual staff and families.

- o Strongly disagree (1)
- o Somewhat disagree (2)
- o Neither agree nor disagree (3)
- o Somewhat agree (4)
- o Strongly agree (5)

Q17 Would you like to share anything else about the effectiveness of the training?

Optional

____

____

Q18 Would you like to share anything else related to your overall experience participating in this training?

Optional

____

____

Q19 Do you have any suggestions to improve this training?

Optional

____

____
